# Phase Noise Analysis of Time Transfer over White Rabbit-Network Based Optical Fibre Links

**DOI:** 10.3390/s24020381

**Published:** 2024-01-08

**Authors:** Neelam, Sithamparanathan Kandeepan, Subhasis Panja

**Affiliations:** 1 School of Engineering, Royal Melbourne Institute of Technology (RMIT), Melbourne, VIC 3000, Australia; s3904453@student.rmit.edu.au; 2 CSIR-National Physical Laboratory, Dr. K. S. Krishnan Marg, New Delhi 110012, India; 3 Academy of Scientific and Innovative Research (AcSIR), Ghaziabad 201002, Uttar Pradesh, India

**Keywords:** White Rabbit, optical fibres, time transfer, phase noise, noise modelling

## Abstract

White Rabbit (WR) is an optical fibre-based time-frequency synchronization technology typically used in timekeeping laboratories for distributing time-frequency signals from a reference clock to distant locations. The accuracy of the received signals at the user end can be affected by random noise processes present in the WR network due to the internal electronic components of WR devices. In this paper, we investigate the presence of random noise processes in the WR network. We then study their statistical properties and model the distribution based on experimentally recorded measurements. According to our study, the probability density function (PDF) follows a Gaussian mixture model (GMM) with varying distribution parameters, and the correlation analysis indicates a strong correlation of the phase noise process over the temporal samples. Furthermore, the developed phase noise models have also been verified by comparing them against additional experimental data. Finally, we present the methodology to generate the phase noise process using computer simulations with the PDF and correlation models developed in this work to help algorithm developers and equipment manufacturers make use of our results.

## 1. Introduction

Time and frequency signal transfer refers to transmitting reference time and frequency signals from reference clocks (such as atomic clocks) to distant locations by employing various kinds of transmission media. Different types of time transfer media affect the accuracy of transferred time–frequency signals differently. The sensitivity of the transmission medium to environmental conditions and noise contributions from the electronic devices used during the time transfer process are the main factors affecting the accuracy of transferred time and frequency signals. Satellite-based time transfer methods such as Common View with Global Navigation Satellite System (CVGNSS) and two-way satellite time and frequency transfer (TWSTFT) are widely used for transferring time and frequency signals and comparing the performance of non-transportable ultra-stable atomic clocks over long distances [1,2]. The accuracy of these methods is affected by the free space environment and faces complex system challenges, high costs, low security, and reliability. Coaxial cables, another medium for transferring time and frequency signals, are not suitable for long distances due to the steep increase in the time transfer error with the length of the link [3]. The increase in time transfer error is caused by propagation loss at high frequencies. In the last few decades, optical fibres have gained attention for transferring accurate and secure time and frequency signals to remote locations. Optical fibres offer several advantages such as large bandwidth, low loss, and strong anti-interference for transferring time and frequency signals over long distances. Moreover, optical fibre links are used by national time laboratories to provide reference time and frequency signals to institutions and compare the remotely located most accurate atomic clocks [4]. Apart from this application, a vast range of other applications can benefit from providing accurate time and frequency signals through optical fibre networks. Examples of such applications include base stations for mobile telecommunication networks, phasor measurement units in electrical power grids, and time-stamping of financial transactions and stock exchanges. In order to compare the performance of ultra-stable clocks, such as optical clocks and Cesium fountains, the stability and accuracy of the established optical fibre links must be at least comparable to or better than these clocks. This necessitates the establishment of highly stable and accurate optical fibre links, as required by national time laboratories and metrological institutes.

More recently, White Rabbit precision time protocol (WRPTP)-based optical fibre links have emerged as a solution for transferring accurate time and frequency signals to remote locations and for comparing the performance of atomic clocks. However, the current stability performance is a few orders of magnitude worse than ultra-stable optical clocks [5]. In order to improve the accuracy and stability of WR-based optical fibre links and meet the requirements of metrological institutes for ultra-stable clock comparison, the phase noise of time transfer over WR-based optical fibre links must be studied.

In this paper, we study and model the phase noise present in time transfer over WRPTP-based optical fibre links. We set up a test environment for a connected WR network with master and slave units to collect real-world data and process them to obtain the phase noise process in the time domain using a commercially available device. The collected data are then analysed to model their distribution in terms of the PDF of the phase noise process. We also study the temporal correlation of the phase noise process and attempt to model this correlation. Finally, we present a complete model for the phase noise random process, followed by the generation of simulated data using the developed model and its verification against the experimentally collected data. To the authors’ knowledge, the phase noise process models adopted in the literature are theory-based in the frequency domain without much treatment of the randomness of the process; moreover, the phase noise process of a network has not yet been treated in the literature. Hence, we consider our work presented here, through the model of the phase noise process in terms of its statistical properties, to be highly valuable to the research community working in this field with the WR network, especially for developing phase noise reduction techniques and algorithms including phase offset estimation for compensation with low phase noise jitter.

The remainder of the paper is organised as follows. In Section 2, we provide a literature review and some relevant background information on WR technology. In Section 3, we present the experimental setup using WR technology and the hardware that was used for the setup. In Section 4 and Section 5, we present the models for the phase noise in terms of the distribution and the temporal correlation, respectively. In Section 6, we show how to use the models that we developed to generate and simulate the phase noise process for WR-based systems. Finally, we present some concluding remarks in Section 7.

## 2. Literature Review and Background

In this section, we present a brief background on WR technology for time and frequency signal transfer, a general review of the phase noise, and a focused review of literature addressing phase noise in WR-based systems.

### 2.1. White Rabbit Technology

**Figure 1:**
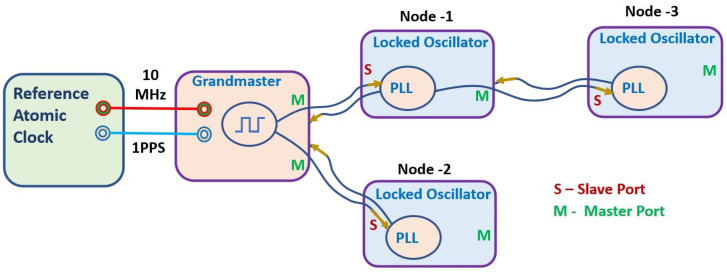
Schematic representation of the WR network.

WR time synchronization technology is an advanced method used to distribute time (1 pulse per second) and frequency (10 MHz) signals from reference clocks to remote locations via optical fibre links [6,7]. This technology was launched at the European Organization for Nuclear Research (CERN) in 2009 and is now available as open source for research and further modifications [8]. The schematic representation of a WR network is shown in Figure 1. According to the figure, the first WR element in the network (WR switch/WR node) is configured in Grandmaster mode by providing reference signals (10 MHz and 1PPS) from the reference atomic clock. The grandmaster then sends synchronization messages to WR elements (Node 1 and Node 2) in its lower hierarchy, establishing the WR link using the master–slave mechanism. After that, following the White Rabbit precision time protocol (WRPTP), slaves (Node 1 and Node 2) are synchronised with the master (Grandmaster). Furthermore, the synchronised slave (Node 1) works as a master for the WR elements that are placed in its lower hierarchy. Here, Node 3 is in the lower hierarchy of Node 1. Therefore, Node 1 acts as the master for Node 3 and sends the synchronization message. Again, following WRPTP, Node 3 is synchronised with Node 1. The same process is repeated in the WR network until all WR elements (WR nodes) are synchronised with the reference clock.

WRPTP includes three key technologies that are used in the WR network to achieve time synchronization: (a) synchronous Ethernet (Sync E), (b) precision time protocol (PTP), and (c) digital dual mixer time difference (DDMTD) [9,10,11]. Along with these key technologies, wavelength division multiplexing (WDM) is used for transmitting and receiving data at different wavelengths through a single strand of optical fibre. Sync E is used to achieve frequency synchronization in the network while PTP and DDMTD are used to measure link delays from master to slave; finally, the link delay is compensated to achieve time synchronization with better-than-nanosecond accuracy. The detailed working principle of WRPTP is explained in reference [12].

There are several types of WR devices, e.g., WR switches, WR nodes, etc. These devices are commercially available. Key WR technologies are incorporated in commercially available WR devices and are used to transfer time with better-than-nanosecond accuracy. Due to the high accuracy, national time laboratories found their application in distributing reference time and frequency signals at distant locations. CSIR-National Physical Laboratory (CSIR-NPL), the National Metrology Institute (NMI) of India, is responsible for distributing reference time and frequency signals to other institutions and distant locations for critical applications. So, at CSIR-NPL, we established a WRPTP-based optical fibre link using the commercially available White Rabbit lite-embedded node (WRLEN) to transfer precise time and frequency signals to distant locations. In this paper, we focus on the analysis and modelling of the phase noise present in WRPTP-based optical fibre links.

### 2.2. Phase Noise

Frequency sources such as oscillators contain noise. These noises are random and nondeterministic, and include thermal noise, shot noise, flicker noise, etc. The presence of this random noise causes time-dependent fluctuations in the phase and amplitude of the signals. These fluctuations are measured and analysed to characterise the stability of the frequency source. The analysis of these noises occurs in both the frequency and time domains. In the frequency domain, these noises are characterised through power spectral density, while in the time domain, they are characterised by Allan deviation, with different types of contributing noises identified by the slopes of Allan deviation vs. averaging time plots [13].

Despite tremendous progress in the past decades in the theory, modelling, analysis, and characterisation of phase noises, certain gaps remain in our understanding of the PDF of random noise processes. In the literature, most reported phase noise studies are conducted in the frequency domain. However, some studies have discussed the PDF for random noises and stated that these are Gaussian in nature. Leaf A. et al. reported a Gaussian PDF for timing jitter in semiconductor mode-locked lasers, where the PDF was estimated by deconvolution of cross-correlation measurements [14]. Y. Yadin et al. reported on phase noise statistics in optical fibre links, stating that the noise distribution aligns well with the Gaussian distribution [15]. J.H. Vukovic et al. also reported on phase noise distribution, stating that intrinsic perturbations originating from transistors in electronic oscillators produce a pure Gaussian distribution, while perturbations from active elements change the Gaussian distribution to an exponential one [16]. However, it was noted that their pure Gaussian distribution for the intrinsic perturbation case could be fitted with a mixture of Gaussian probability density functions.

### 2.3. Phase Noise in White Rabbit Systems

WRPTP-based time transfer links include reference clock sources, such as atomic clocks, optical fibres, small form-factor pluggable transceivers, and WR-compliant devices like WR switches and WR nodes, which establish communication within the WR network to transfer precise time and frequency signals over long distances. In addition to the essential components of a conventional WR network, phase-compensating devices are included to compensate for phase variations and improve time synchronization accuracy. Measuring devices, such as time interval counters, are an indispensable part of time transfer experimental setups. The internal electronic components of devices used in WR-based time transfer links contribute random noise to time delay measurements, affecting the time transfer accuracy. Phase noise contributions from internal components of a WR switch, such as digital dual mixer time difference detectors and gigabit Ethernet transceivers, have been reported by M. Rizzi et al. [17]. They stated that both phase detectors and transceivers have a similar phase noise contribution, which is dominated by flicker phase modulation. M. Rizzi et al. have also reported the contribution of 1 Gbps fibre optic transceivers to overall uncertainty, stating that it is practically negligible compared to other uncertainties in the system [18]. P. Li et al. studied the noise-contributing factors and developed a Kalman filter to improve time synchronization accuracy [19]. So far, the phase noise contributions of different electronic components in the WR system have been analysed and reported in the frequency domain. The statistical phase noise analysis of noise present in WR-based systems has not yet been reported. To reduce the impact of random noise originating from electronic components, like phase detectors, Ethernet transceivers inside the WR nodes, and electronic components of phase-compensating devices in the WR network, and to find better solutions to improve time transfer accuracy over WR network-based optical fibre links, the statistical properties of the noises must be characterised. In this article, we present a statistical analysis of the random noise in time transfer over WR network-based optical fibre links.

## 3. Experimental Setup for Phase Noise Measurements

In this section, we present an experimental setup for studying the phase noise present in time transfer over WR network-based optical fibre links. To collect, analyse, and model the phase noise, WR network-based optical fibre links were established. The WR network setup consists of three White Rabbit Lite Embedded Nodes (WRLENs, Seven Solutions), a pair of single-mode optical fibre spools with a core diameter of 10 micrometres, and bidirectional small form-factor pluggable transceivers (SFPs). A Cesium (Cs) atomic clock acts as the reference clock in the WR network.

**Figure 2:**
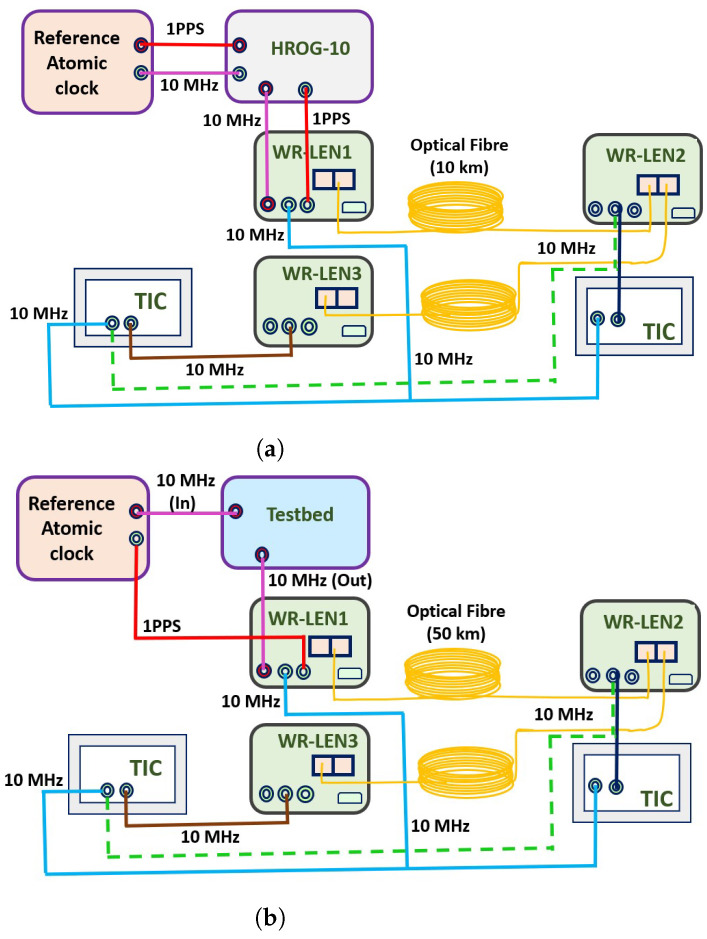
Schematic diagram of the experimental test setups to study the phase noise of time transfer over the WR network. (**a**) Experimental setup A with HROG-10; (**b**) experimental setup B with the testbed.

In WR networks, phase offsets are usually compensated to improve the accuracy of the time transfer. Therefore, we introduce a phase and frequency offset generator (HROG-10, spectradynamics) to compensate for the phase offsets in the network. However, in the study presented in this paper on phase noise analysis, we have not enabled the phase compensation algorithm but have only included the HROG-10 in the network. Such an approach was adopted to model a typical WR network with a phase compensator, helping to study how the phase compensator hardware unit itself contributes to the phase noise of the WR network. Therefore, in order to study the noise contribution due to the phase compensator in the WR network, the HROG-10 was included. The schematic representation of the experimental setup is shown in Figure 2a.

The reference Cs atomic clock signals were provided to WRLEN1 through HROG-10. WRLENs were used to transfer time and frequency signals and could be configured in ‘Grandmaster(GM)’, ‘Master’, and ‘Slave’ modes. The functioning of WRLENs in these configurations is briefly summarised below.

- Grandmaster (GM): In this mode, external reference signals from a highly stable reference clock are required to lock the inbuilt oscillator of the WRLEN with reference clock signals. In the GM mode, both ports of the WRLEN work as masters.
- Master: In this mode, no external reference signals are needed, and the WRLEN’s inbuilt oscillator runs freely. Both ports of the WRLEN work as masters in this mode.
- Slave: In this mode, no external reference signals are needed, and one port of the WRLEN acts as the slave while the other acts as the master. In the present study, in ‘Slave’ mode, the first port of the WRLEN acts as the slave and the second port as the master, as shown in Figure 2a.

As shown in Figure 2a, i.e., experimental setup A, WRLEN1 is configured in GM mode by providing 10 MHz and 1 pulse per second (1PPS) reference signals from a Cs atomic clock through a phase compensator (HROG-10). WRLEN2 and WRLEN3 are in ‘Slave’ mode. WRLEN2, representing a remotely located clock, is connected to WRLEN1 through optical fibre. Similarly, WRLEN3 is connected to WRLEN2 with another optical fibre of the same length and is collocated with WRLEN1. The fibre spools are 10 km in length and the SFP transceivers operate at 1490/1310 nm. Time interval counters (TIC, Keysight 53230A) are used to measure the phase offset between different WRLENs.

Similarly, in experimental setup B, as shown in Figure 2b, the noise contribution of the testbed is studied by replacing the commercially obtained phase compensator HROG-10 with an in-lab integrated testbed. The testbed is a cost-effective alternative to the commercial HROG unit, and its design, development, and key features are briefly explained in Section 3.2 WRLEN1 is configured in GM mode with a 10 MHz reference signal from the testbed, while the 1PPS signal is directly provided from the reference atomic clock. WRLENs are connected using optical fibres in the same manner as in test setup A. The optical fibre spools are 50 km in length, and the SFP transceivers operate at 1550/1310.

All three WRLENs in the WR network follow WRPTP and are synchronised with the reference Cs atomic clock. In this way, time and frequency signals are transferred from the reference Cs atomic clock to distant locations. However, the internal components of the electronic devices used in the WR network, such as phase detectors, transceivers inside the WRLENs, and other fundamental electronic components of phase-compensating devices, contribute random noise and cause variations in the signal’s phase.

### 3.1. Data Collection and Processing

In order to study random noise in the time and frequency signal transfer over WR network-based optical fibre links, phase variations of 10 MHz signals obtained at the outputs of different WRLENs were recorded using a commercially available Keysight 53230A, 350 MHz universal frequency counter/timer.

The important features of the time interval counter and the data collection process for all experiments presented in this paper are discussed below.

**The time interval counter**: The Keysight 53230A time interval counter (TIC) offers features for measuring time interval, phase, frequency, frequency ratio, and period [20]. These measurements can be recorded in its internal memory or an external memory device. This TIC model allows the “Gate Time” to be set in a range from a minimum of 1 μs to a maximum of 1000 s. Control over the “Gate Time” to set the required time period for recording each data point enables setting the Gate Time at 1 ms and 1 s.

In this study, we recorded phase variations between the 10 MHz output signals of WRLENs connected through optical fibres within the WR network using the TIC. The “Time Interval” key of the TIC includes options for measuring the time interval, rise/fall time, pulse width, duty cycle, phase, and single period. The phase offset between 10 MHz output signals of WRLENs was recorded by selecting the “phase” option and setting the required gate time. The 10 MHz output signals of WRLEN1 (light blue) and WRLEN2 (dark blue) were fed to the TIC input ports to measure and record phase variations between the WRLENs. Similarly, phase offsets between WRLEN2 (10 MHz output, dotted green) and WRLEN3 (10 MHz output, brown) and between WRLEN1 (10 MHz output, light blue) and WRLEN3 (10 MHz output, brown) were recorded to study the phase variation properties, as shown in the schematic diagram in Figure 2.

The phase variations recorded in these cases exhibit random variations; therefore, a statistical treatment for the analysis was adopted. The temporal variation of the phase is treated as a statistical random process, and we term the phase variations as the phase noise process $\phi_{n}$ in this paper. Different sets of data, namely, phase offsets between WRLEN1 and WRLEN2, WRLEN2 and WRLEN3, and WRLEN1 and WRLEN3, have been recorded in the internal memory of TIC in a “.csv file” format and then copied to an external device to analyse the random noise properties offline.

### 3.2. Testbed Components, Design, and Features

As mentioned previously, and shown in Figure 2b for experimental setup B, HROG-10 is replaced with a low-cost custom-designed testbed for phase-offset compensation. In this section, we describe the testbed setup details. The testbed comprises a time-to-digital converter evaluation module (TDC 7200 EVM) and a direct digital synthesizer (DDS AD9912 evaluation module). The important features of these modules have been summarised below.

**Figure 3:**
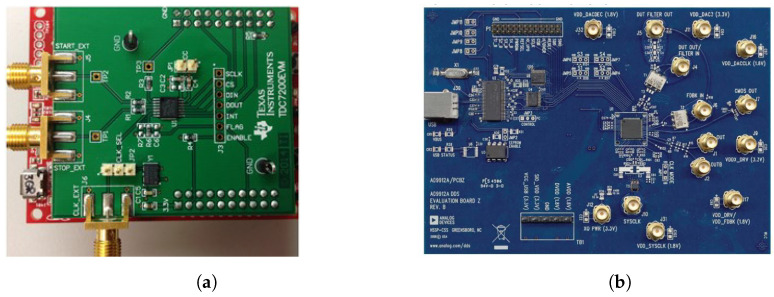
Time-to-digital converter (TDC) 7200 and AD9912 direct digital synthesizer evaluation module. (**a**) TDC 7200 EVM; (**b**) AD9912 DDS board.

**The time-to-digital converter, TDC-7200 EVM**: TDC 7200 EVM, as shown in Figure 3a [21], is a time-to-digital converter evaluation module that measures the time offset between electronic 1PPS signals fed to its input channel START and STOP ports. It can measure only positive time offsets between the 1PPS signals in the range of 12 ns to 8 ms with 55 ps resolution and can be operated with its graphical user interface. It also provides the feature of using an external reference clock signal to avoid errors in measurements due to any drift in the internal crystal oscillator frequency.

**The direct digital synthesizer ad9912 evaluation module**: AD9912 is a low noise, 14-bit DDS clock synthesizer, as shown in Figure 3b. It has both square and sinusoidal output signals. The frequency of the output sinusoid generated by the DDS is determined by a frequency tuning word (FTW), which is a digital value. The relative phase of the sinusoid can be controlled numerically using the phase offset function of the DDS through a programmable 14-bit value. Its resolution is 0.022 degrees and can be used to generate positive as well as negative phase offsets. It has a system clock phase-locked loop (PLL), and the system clock input accepts either a crystal (internal) or external reference clock [22].

**Figure 4:**
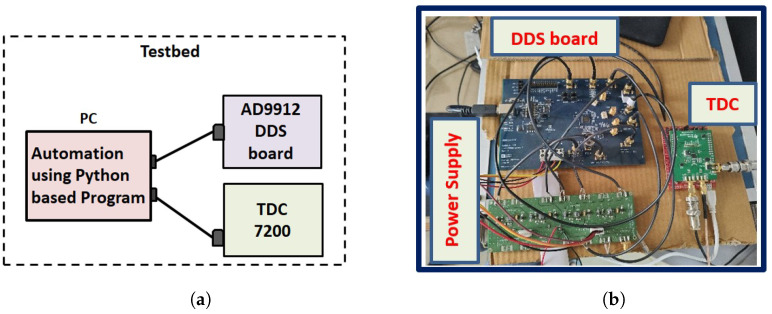
Testbed design to introduce automated dynamic phase offsets to compensate for phase variations. (**a**) Testbed architecture. (**b**) Real image of the testbed.

**The testbed design**: The testbed was developed by interfacing and integrating TDC 7200 EVM and AD9912 DDS modules using a Python programming language to automate the phase compensation process in the WR network. The testbed architecture and its real image are shown in Figure 4.

The DDS board (AD 9912 EVM) power supply requirements (1.8 V and 3.3 V) were fulfilled through the power supply board and interfaced by connecting it with a computer using a USB cable. The TDC board (7200 EVM) does not require an external power supply; it uses a supply voltage of 2 to 3.6 V from the computer through a USB cable. After that, both evaluation modules were interfaced and integrated to measure variations in time delays and introduce automated phase corrections (Figure 4). In this study, we investigated the noise contribution of the testbed by including it in the WR network-based time transfer link without using its automated phase compensation features, as shown in Figure 2b.

## 4. Phase Noise Modelling: Statistical Distribution

In this section, we present the statistical analysis of the phase noise process for time transfer over the WR network-based optical fibre link. Phase noise was analysed using the recorded phase offset data between the different WRLENs. Upon observing the phase noise data, a running average (mean) of the phase noise process was subtracted from it, to achieve a close-to-zero mean process. The treatment of the running average of the phase process and the corresponding variation of it over time is beyond the scope of this paper and is expected to be looked at in future work. In this paper, we purely focus on the phase noise process where the running average was subtracted.

Based on the analysis of the phase noise data, we adopted a Gaussian mixture model (GMM) to model the probability density function of the phase noise process with different mixture components, *k*. The PDF of the GMM model is represented as a weighted sum of *k* components of Gaussian densities, given by

(1) $$p\left(\phi_{n}\right)=\sum_{i=1}^{k}w_{i}g\left(\phi_{n}|\mu_{i},\sigma_{i}\right)$$

where $\phi_{n}$ is the phase noise process, *w*_i_, i=1,2…k, are the mixture weights, and $g(\phi_{n}|\mu i,$σi),i=1,2,…,k are the Gaussian mixture densities with a mean $\mu_{i}$ and covariance matrix $\sigma_{i}$. In our analysis, the GMM parameters, such as the mean, standard deviation, and respective weights of the phase noise process for the different data sets are estimated using the iterative expectation–maximization (EM) algorithm, which is a well-known estimation method in the field of statistics.

The models that we present in the following section for the statistical phase noise distribution are categorised into two cases, case 1: the WR network setup with the HROG-10 unit, and case 2: the WR network setup with the custom-designed testbed unit. In addition to the different cases mentioned above, we also present models by varying the gate time *T*. It should be noted that in all the cases mentioned above (case 1 and case 2), including the variation of the gate time *T*, the distribution follows a GMM model with different model parametric values.

### 4.1. Phase Noise Distribution Analysis for Experimental Setup A with HROG-10

The system diagram for experimental setup A with HROG-10 is shown in Figure 2a. Here, HROG-10 was used to study its noise contribution within the WR network. The reference signal from the reference Cs atomic clock is provided to WRLEN1 through the HROG-10. The phase offsets between different WRLENs, such as between WRLEN1 and WRLEN2, WRLEN2 and WRLEN3, and WRLEN1 and WRLEN3, were measured and different data sets were created by varying the “Gate Time (T)”. The time window *T* is the same as the data observation window; that is, it represents the duration of the sample set collected for the phase process analysis. The phase difference (offset) was recorded at two different gate times, T=1 ms and T=1 s, and the corresponding phase noise analysis and modelling in terms of the statistical distribution are provided below.

#### 4.1.1. Data Collection with Gate Time: T=1 ms

For experimental setup A, with a gate time of T=1 ms, three sets of data, Data Set 1, Data Set 2, and Data Set 3, were collected as reported in this section.

**Figure 5:**
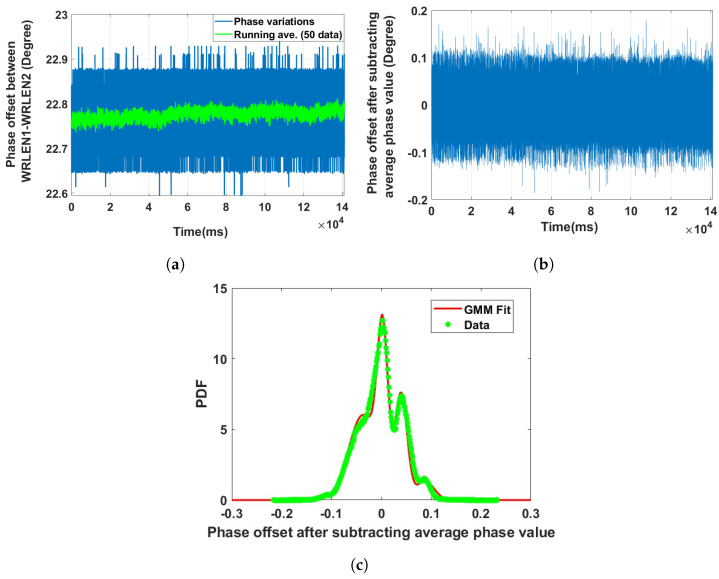
Noise analysis of the phase offset between WRLEN1 and WRLEN2 for test setup A with HROG-10. (**a**) Phase offset between WRLEN1 and WRLEN2; (**b**) phase offset between WRLEN1 and WRLEN2 after subtracting the average phase value; (**c**) probability density function of the phase noise between WRLEN1 and WRLEN2.

**Data set 1—phase offset between WRLEN1 and WRLEN2:** In this data set, the phase difference between WRLEN1 and WRLEN2 was recorded using the TIC at T=1 ms. The nodes WRLEN1 and WRLEN2 were connected with a 10 km optical fibre. The phase offset variations shown in Figure 5a indicate that peak-to-peak phase variations are within 0.3 degrees. The running average of the phase difference was calculated by averaging over 50 data points and subtracted from the original phase difference to form the phase difference data for the modelling, as shown in Figure 5b. The PDF of the phase noise between WRLEN1 and WRLEN2 was a GMM and fitted with 5 mixing components, as shown in Figure 5c. The estimated GMM parameter is tabulated in Table 1. The component proportion of GMM parameters indicates that out of these five mixing components, only three components have noticeable contributions, with *w*_i_ = 0.43, 0.28, and 0.22, as shown in Figure 5c.

**Figure 6:**
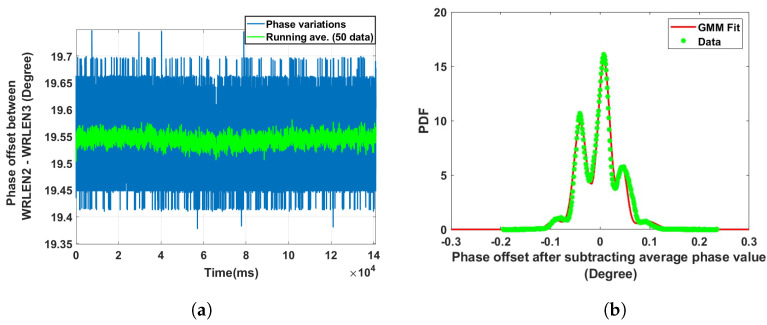
Noise analysis of the phase offset between WRLEN2 and WRLEN3 for test setup A with HROG-10. (**a**) Phase offset between WRLEN2 and WRLEN3; (**b**) probability density function of the phase noise between WRLEN2 and WRLEN3.

**Data set 2—phase offset between WRLEN2 and WRLEN3:** In this data set, the phase difference between WRLEN2 and WRLEN3 was recorded at T=1 ms with a 10 km optical fibre. The peak-to-peak phase variation lies within 0.3 degrees, as shown in Figure 6a. The phase difference between the WRLEN2 and WRLEN3 pdf consists of five mixing components. However, only three mixing components have significant contributions, with *w*_i_ = 0.32, 0.48, and 0.13, as shown in Figure 6b.

**Figure 7:**
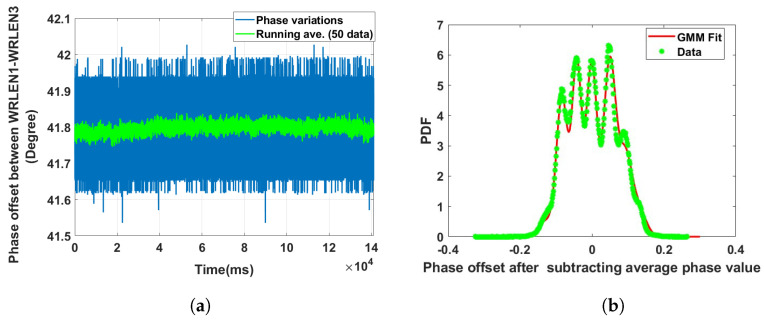
Noise analysis of the phase offset between WRLEN1 and WRLEN3 for test setup A with HROG-10. (**a**) Phase offset between WRLEN1 and WRLEN3; (**b**) probability density function of the phase noise between WRLEN1 and WRLEN3.

**Data set 3—phase offset between WRLEN1 and WRLEN3:** In this data set, the phase difference between WRLEN1 and WRLEN3 was recorded at T=1 ms. In the experimental setup, as shown in Figure 2a, WRLEN2 is connected to WRLEN1 with a 10 km fibre. Similarly, WRLEN3 is connected to WRLEN2, and there was no direct connection between WRLEN1 and WRLEN3. However, WRLEN3 is connected to WRLEN1 through WRLEN2, following the daisy-chain configuration of the WR network. So, in this case, all three WRLENs and both optical fibres, each 10 km long, contribute to the measured phase variations. The peak-to-peak phase variations lie within 0.4 degrees, which is more than the observed phase variations in the previous cases, as shown in Figure 7a. The PDF of the phase noise between WRLEN1 and WRLEN3 can be fitted with a GMM PDF having 8 mixing components. The estimated GMM parameters, shown in Table 1, indicate that out of eight mixing components, only four mixing components with $w_{i}$ = 0.17, 0.18, 0.22, and 0.26 have significant contributions, as shown in Figure 7b. Compared to the previous two data sets, it was found that due to the increase in the number of WRLENs from 2 to 3, and the length of fibre from 10 km to 20 km during the data set measurements, the phase variations increased from 0.3 degrees to 0.4 degrees, and the number of significant mixing components increased from 3 to 4 in the GMM PDF. This observation indicates that phase noise is contributed by each WRLEN and the increase in fibre lengths.

#### 4.1.2. Data Collection with Gate Time: T=1 s

We repeated the setup A experiment with HROG-10 and recorded phase variations for a longer period of time at T=1 s to study the phase noise behaviour. For T=1 s, we further recorded two more sets of data, Data set 4 and Data set 5, as reported below.

**Figure 8:**
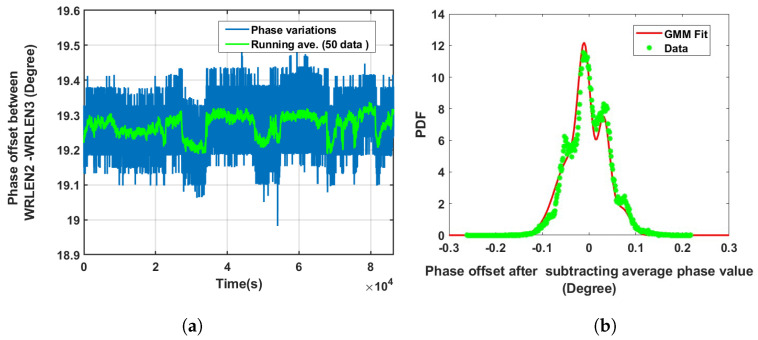
Noise analysis of the phase offset between WRLEN2 and WRLEN3 at T=1 s for test setup A with HROG-10. (**a**) Phase offset between WRLEN2 and WRLEN3; (**b**) probability density function of the phase noise between WRLEN2 and WRLEN3.

**Data set 4—phase offset between WRLEN2 and WRLEN3:** In this data set, phase variations between WRLEN2 and WRLEN3 were recorded for a period of 24 h at T=1 s with 10 km fibres, and phase noise behaviour was studied. The phase variations between WRLEN2 and WRLEN3 are shown in Figure 8a. The peak-to-peak phase variations lie within 0.35 degrees. Phase noise data were processed in a similar way as they were estimated in previous cases by subtracting the running average. The PDF of the phase noise is shown in Figure 8b. The estimated GMM PDF parameters indicate that GMM consists of six mixing components. However, out of six, only three mixing components with $w_{i}$ = 0.34, 0.32, and 0.24 have significant contributions, as shown in Figure 8b and Table 1. Phase variations and the GMM PDF of the phase noise between WRLEN2 and WRLEN3 at T=1 s were compared with the phase variations and PDF of the phase noise between these nodes at T=1 ms. It was found that peak-to-peak variations slightly increased by 0.05 degrees over a period of 1 day of measurement, while the number of significant mixing components in the GMM PDF remained the same.

**Figure 9:**
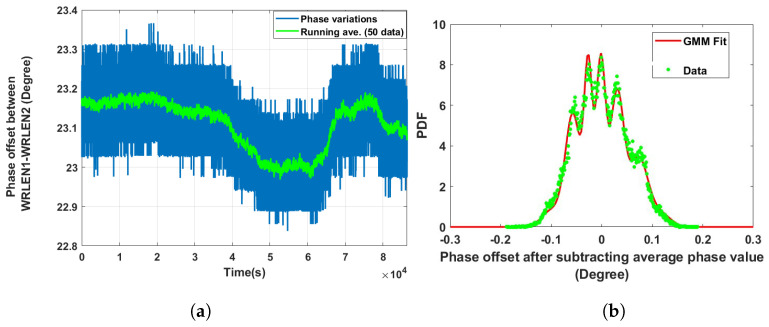
Noise analysis of the phase offset between WRLEN1 and WRLEN2 at T=1 s for test setup A with HROG-10. (**a**) Phase offset between WRLEN1 and WRLEN2; (**b**) probability density function of the phase noise between WRLEN1 and WRLEN2.

**Data set 5—phase offset between WRLEN1 and WRLEN2:** In this data set, the phase difference between WRLEN1 and WRLEN2 was recorded at T=1 s with a 10 km fibre, over a period of one day. Data set 4, i.e., the phase offset between WRLEN2 and WRLEN3, and data set 5, i.e., the phase offset between WRLEN1 and WRLEN2, were recorded simultaneously utilising two time interval counters. So, the ambient temperature conditions for both cases were the same. However, by analysing and comparing the phase variations of both data sets, it is observed that in data set 5, peak-to-peak phase variation was 0.4 degrees, shown in Figure 9a, while for data set 4, it was 0.35 degrees. The phase noise GMM pdf of data set 5 consists of seven mixing components. The calculated GMM parameters of data set 5 show that out of seven mixing components, five mixing components with $w_{i}$ = 0.21, 0.15, 0.19, 0.22, and 0.16 have a significant contribution, as shown in Figure 9b. For data set 4, the number of significant mixing components was only three. These observations suggest that over a long duration, under the same ambient temperature conditions, with the same length of fibres, and for the same time period, the phase noise between WRLEN1 and WRLEN2 in the GMM PDF has more mixing components compared to the phase noise between WRLEN2 and WRLEN3 in the GMM PDF. The increase in the number of mixing components, as well as in peak-to-peak phase variations, can be related to the significant temperature sensitivity of WRLEN1 in Grandmaster mode [23,24]. In Grandmaster mode, the continuous operation of the phase-lock loop to lock the inbuilt oscillator with the reference atomic clock signals makes it sensitive to ambient temperature variations. The literature reports that WRLENs in Grandmaster mode are significantly sensitive to temperature variations, while in “Slave” mode, the temperature sensitivity of WRLENs is not considerable. As in data set 5, WRLEN1 is configured in Grandmaster mode, while in data set 4, both WRLENs, i.e., WRLEN2 and WRLEN3, are in ’Slave mode’. So, in data set 5, WRLEN1 in GM mode leads to an increase in the number of GMM mixing components compared to data set 4. With data set 1, i.e., the phase offset between WRLEN1 and WRLEN2 at T=1 ms, this behaviour was not noticeable because the data were recorded for a very short period of time, i.e., for 30 min only. Due to this, the number of significant mixing components and phase variations for the phase offset recorded at T=1 ms for data set 1 and data set 2 were the same.

### 4.2. Phase Noise Distribution Analysis for Experimental Setup B with the Testbed

In this case study, we replaced the commercial phase compensator HROG-10 with an in-house developed testbed to study the phase noise behaviour of time transfer over a WR network. The reference signal from the reference Cs atomic clock was provided to WRLEN1 through the testbed to configure WRLEN1 in Grandmaster mode and to study the impact of the testbed on phase variations. The length of the optical fibre was 50 km, and the transceivers were operating at 1550/1310 nm. The phase noise was analysed and recorded following a similar process to previous cases. The phase offset between different WRLENs was recorded at a gate time of T=1 ms, and the analysis is presented below.

#### Data Collection with Gate Time: T=1 ms

For experimental setup B, with a gate time of T=1 ms, three sets of data, i.e., Data set 6, Data set 7, and Data set 8, were collected as reported in this section.

**Figure 10:**
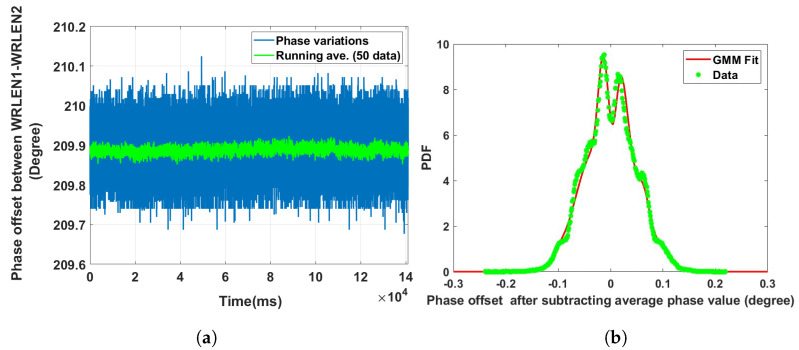
Noise analysis of the phase offset between WRLEN1 and WRLEN2 at T=1 ms for test setup B with the testbed. (**a**) Phase offset between WRLEN1 and WRLEN2; (**b**) probability density function of the phase noise between WRLEN1 and WRLEN2.

**Data set 6—phase offset between WRLEN1 and WRLEN2:** In this data set, phase variations between WRLEN1 and WRLEN2 were recorded utilising TIC at T=1 ms with 50 km fibres as shown in Figure 2b. The peak-to-peak phase variations lie within 0.3 degrees, as shown in Figure 10a. The PDF analysis of the phase noise for test setup B with the testbed shows that the PDF again fits with a GMM, as observed for test setup A with HROG-10. The GMM PDF of the phase noise between WRLEN1 and WRLEN2 consists of 8 mixing components. However, the estimated GMM parameters shown in Table 2 indicate that only two mixing components (with $w_{i}$ = 0.235 and 0.197) have significant contributions, as shown in Figure 10b. The number of significant mixing components in data set 1, i.e., the phase noise between WRLEN1 and WRLEN2 at T=1 ms for test setup A with HROG-10, is 3, while for the current data set, it is 2. The change in mixing components with noticeable contributions in the PDF from 3 to 2 at T=1 ms is caused due to the replacement of HROG-10 with the testbed.

**Figure 11:**
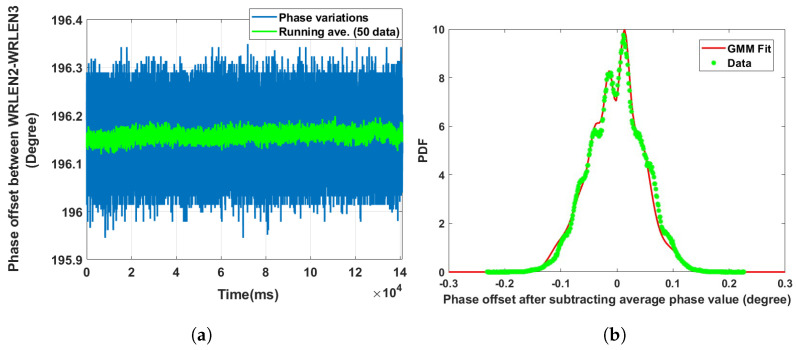
Noise analysis of the phase offset between WRLEN2 and WRLEN3 for test setup B with the testbed. (**a**) Phase offset between WRLEN2 and WRLEN3; (**b**) probability density function of the phase noise between WRLEN2 and WRLEN3.

**Data set 7—phase offset between WRLEN2 and WRLEN3:** In this data set, the phase difference between WRLEN2 and WRLEN3 is recorded at T=1 ms with a 50 km fibre. The peak-to-peak phase variation lies within 0.3 degrees, as shown in Figure 11a. The GMM pdf for the estimated phase noise between WRLEN2 and WRLEN3 fits with the eight mixing components shown in Table 2. However, the GMM parameters show that only two components (with $w_{i}$ = 0.20 and 0.22) have significant contributions, as shown in Figure 11b. The mixing component for the phase noise between WRLEN2 and WRLEN3 PDF is the same as between WRLEN1 and WRLEN2, as observed for the previous case at T=1 ms with 50 km. This observation confirms that phase variations and mixing components, which contribute significantly in GMM PDF for the phase noise recorded between WRLEN1 and WRLEN2 and between WRLEN2 and WRLEN3 at T=1 ms, remain the same. WRLEN1, which is configured in Grandmaster mode, does not contribute any noticeable phase variations for short periods i.e., for nearly 30 min.

**Figure 12:**
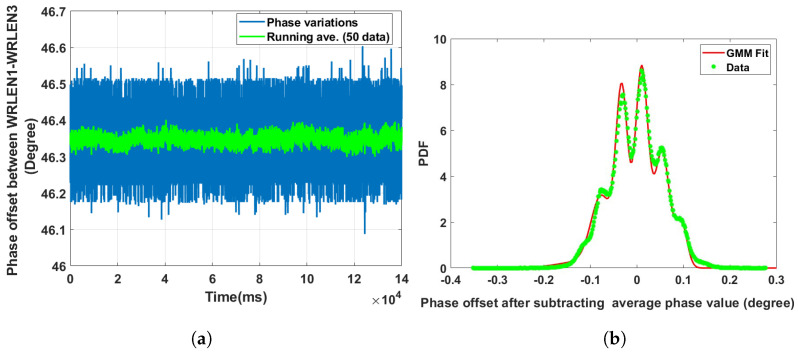
Noise analysis of the phase offset between WRLEN1 and WRLEN3 for test setup B with the testbed. (**a**) Phase offset between WRLEN1 and WRLEN3; (**b**) probability density function of the phase noise between WRLEN1 and WRLEN3.

**Data set 8—phase offset between WRLEN1 and WRLEN3:** In this data set, the phase difference between WRLEN1 and WRLEN3 was recorded at T=1 ms. The WRLEN3 was connected to WRLEN1 through WRLEN2 by following the WR network daisy-chain configuration. So, all three WRLENs contribute to phase variations between WRLEN1 and WRLEN3, and the total length of fibre is 100 km. The peak-to-peak phase variations lie within 0.3 degrees, as shown in Figure 12a. The PDF of the phase noise between WRLEN1 and WRLEN3 fits with GMM and has six mixing components. However, according to the estimated GMM parameters, only four mixing components with $w_{i}$ = 0.16, 0.24, 0.30, and 0.20 have significant contributions, as shown in Figure 12b. In data set 3, i.e., in the phase offset between WRLEN1 and WRLEN3 for test setup A with HROG-10, we found that the number of mixing components was greater than the mixing components for data set 2 and data set 3. Similarly, for the current data set, the number of significant mixing components, which is 4, is greater than the significant mixing components for data set 6 and data set 7, which is 2.

Hence, analysing the obtained results, we conclude that, over a short period at T=1 ms, peak-to-peak phase variations lie within 0.3 degrees for both test setups, i.e., test setup A and test setup B, and the distribution fits with a GMM model. Moreover, the number of significant mixing components in GMM PDF for the phase offset between WRLEN1 and WRLEN3 was greater than the mixing components in the phase noise between WRLEN1 and WRLEN2 and between WRLEN2 and WRLEN3 for both test setups. It has also been observed that the phase noise GMM PDF consists of the same number of mixing components if the number of WRLENs and fibre lengths is the same, and the phase offset is recorded for short time durations, i.e., at T=1 ms. The increase in the number of WRLENs and the length of fibre during the measurement of a particular data set’s phase offset leads to an increase in the number of mixing components in the phase noise GMM PDF. The temperature sensitivity of WRLEN1 in Grandmaster mode affects the phase variations and contributes to an increase in the number of mixing components in the GMM PDF, particularly when the phase offset between WRLEN1 and WRLEN2 is recorded over a long period of time.

## 5. Phase Noise Modelling: Autocorrelation

The correlation or pattern repetition in time series data is studied to measure the strength of a linear relationship between two variables. To understand the correlation of the phase noise process of time transfer over a WR network, as studied in this paper, the autocorrelation functions (ACFs) of different phase noise data sets are analysed and modelled. The normalised ACF of a particular phase noise process $\phi_{n}\left(t\right)$ is defined by

(2) $$R_{\phi_{n}}\left(\tau \right)=\frac{1}{R_{0}}\int_{-\infty }^{\infty }\phi_{n}\left(\tau \right)\phi_{n}\left(t-\tau \right)$$

where τ is the lag of $R_{\phi_{n}}\left(\tau \right)$ and $R_{0}=R_{\phi_{n}}\left(0\right)$. The ACF analysis and modelling for different data sets of the phase noise process are presented in the following sections.

### 5.1. ACF of the Phase Noise for Test Setup A with HROG-10

**Figure 13:**
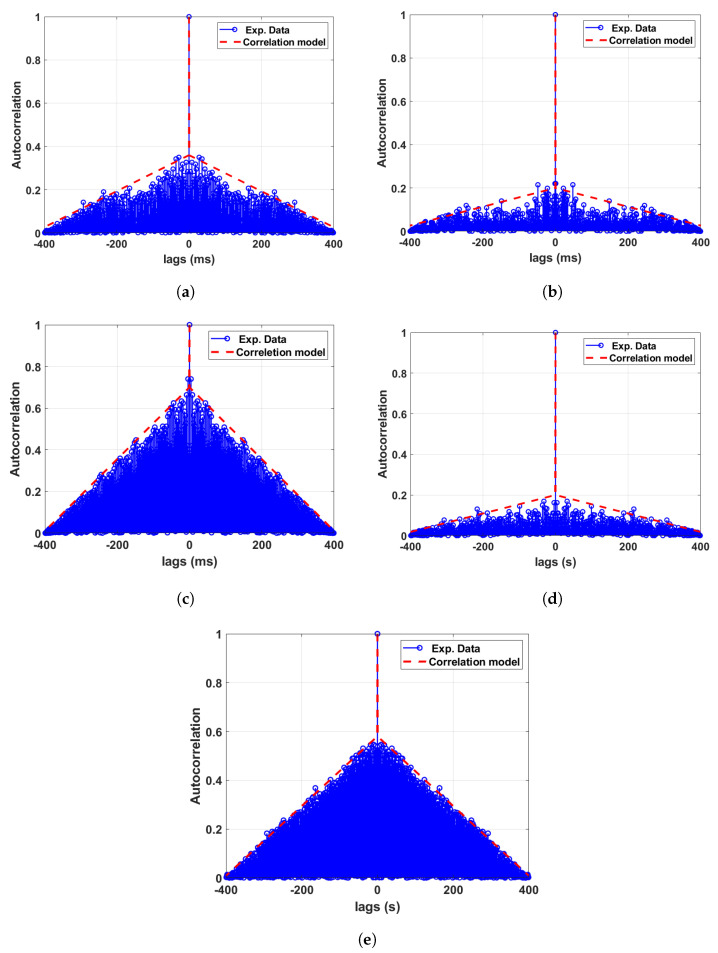
Autocorrelation analysis of the phase noise between different WRLENs for the data sets recorded with test setup A. (**a**) Autocorrelation function of the phase noise between WRLEN1 and WRLEN2; (**b**) autocorrelation function of the phase noise between WRLEN2 and WRLEN3; (**c**) autocorrelation function of the phase noise between WRLEN1 and WRLEN3; (**d**) autocorrelation function of the phase noise between WRLEN2 and WRLEN3 at a 1 s interval; (**e**) autocorrelation function of the phase noise between WRLEN1 and WRLEN2 at a 1 s interval.

For experimental setup A, the ACF of the phase noise process for all respective data sets was analysed, and the corresponding results for the magnitude of the ACF are shown in Figure 13. The figure also shows the model that we adopt for the ACF, where the model itself is presented in detail in a later section. From the results, we observe that all data sets follow the same trend, where the phase noise process $\phi_{n}\left(t\right)$ shows a significant correlation in time. However, the magnitude of the correlation coefficient at shorter lags differs for different cases (data sets), as observed in Figure 13. It is worthwhile to note here that all WRLENs are driven by a single source, which is the Cs atomic clock. WRLEN1 in the WR network is configured in Grandmaster mode by providing reference signals from the Cs atomic clock.

### 5.2. ACF of the Phase Noise for Test Setup B with the Testbed

**Figure 14:**
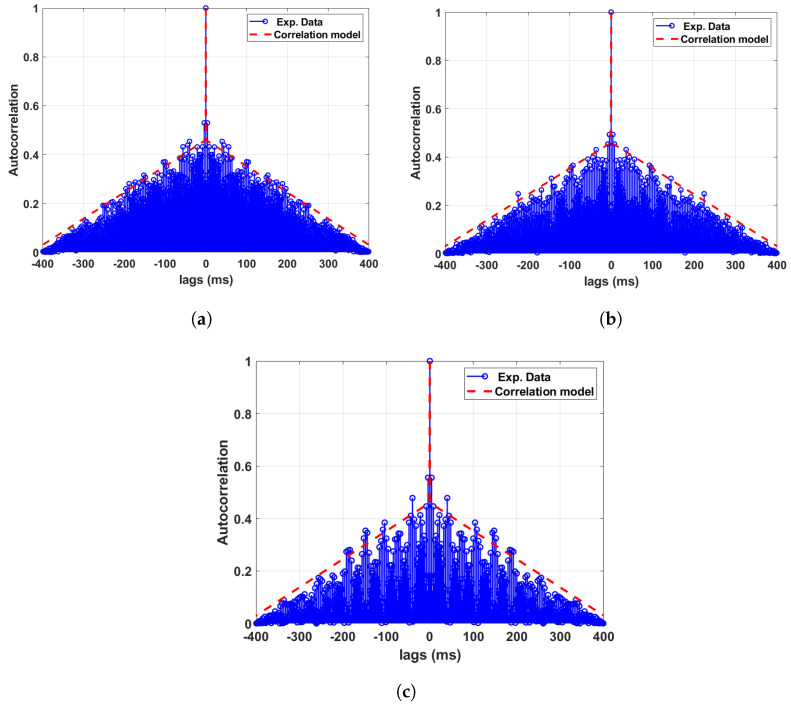
Autocorrelation analysis of the phase noise between different WRLENs for the data sets recorded with test setup B. (**a**) Autocorrelation for the phase noise between WRLEN1 and WRLEN2; (**b**) autocorrelation for the phase noise between WRLEN2 and WRLEN3; (**c**) autocorrelation for the phase noise between WRLEN1 and WRLEN3.

Similar to the ACF analysis for setup A, we also study the ACF of the phase noise process for experimental setup B. The results of the magnitude of the ACF for all three data sets related to setup B are shown in Figure 14. The trend of the ACF for experimental setup B is found to be very similar to that of setup A. The figures also show the ACF model, which we will present in the next section.

### 5.3. Modelling of the Phase Noise Autocorrelation Functions

Modelling the ACF of any random process is quite challenging; the ACF can usually have different long-term and short-term behaviours typically following exponentially decaying oscillatory behaviours. In our analysis, we adopt a simplified yet convincing model, as presented in this section. We also verify the proposed simplified model for the magnitude of the ACF $|R_{\phi_{n}}\left(\tau \right)|$ of the phase noise process by comparing it against the chosen data sets. It is well understood that the models presented in this paper are not globally unique; however, the intention is to provide a model that reflects real-world processes, which researchers and engineers can adopt for their own respective research and development works.

By observing the sample ACF calculated using the recorded data sets for $\phi_{n}$, we identify that $|R_{\phi_{n}}\left(\tau \right)|$ can be fitted with the following model.

(3) $$|R_{\phi_{n}}\left(\tau \right)|=\left\{\begin{array}{cc} 1 & \mathrm{for}\;\tau =0\;\\ (-\frac{\tau }{a})+b & \mathrm{for}\;\tau \neq 0\; \end{array}\right.$$

where *a* and *b* are real constants and their values for different data sets are given in Table 3. The modelled ACFs are shown in Figure 13 and Figure 14, with the dotted red line corresponding to the model parameters given in Table 3.

## 6. Phase Noise Generation Using the Modelled PDF and ACF

The proposed models for the PDF and the ACF, as presented in Table 1, Table 2 and Table 3, are further verified by generating the corresponding phase noise process using the modelled PDF and ACF. To perform the verification and to illustrate how to simulate the phase noise process studied in this paper, this section presents the generation of random phase noise using the models presented in the previous sections. Once the model’s data (i.e., phase noise) are generated, we then compare them with the experimental data for our final verification of the proposed model presented in this paper. The generation of phase noise is a two-step process well-known in the fields of statistics and data analytics. In the first step, we generate the phase noise process according to the modelled PDF, and in the second step, we manipulate the generated random process to match the modelled ACF. We further describe the two steps for generating the phase noise process below.

### 6.1. Phase Noise Generation Using the Modelled PDF

The ‘Inversion Method’ is the most commonly used technique for generating sample values of random variables using a prescribed PDF. The basic steps to generate a random variable—and, hence, the random process, using this method—are summarised as follows. The ’inversion’ or ’inverse transformation’ method for generating random variables takes random samples from a uniform distribution **U** between 0 and 1 to generate the target cumulative distribution function (CDF) of the phase noise $F(\phi_{n})$ that is continuous and strictly increasing, such that $0<F(\phi_{n})<1$. If we let $F^{-1}$ denote the inverse of the CDF function, then the inverse transformation algorithm is given by the two sub-steps:

**Step 1.1**: generate *u*∼**U** (0,1)

**Step 1.2**: Return $\phi_{n}=F^{-1}\left(u\right)$

**Figure 15:**
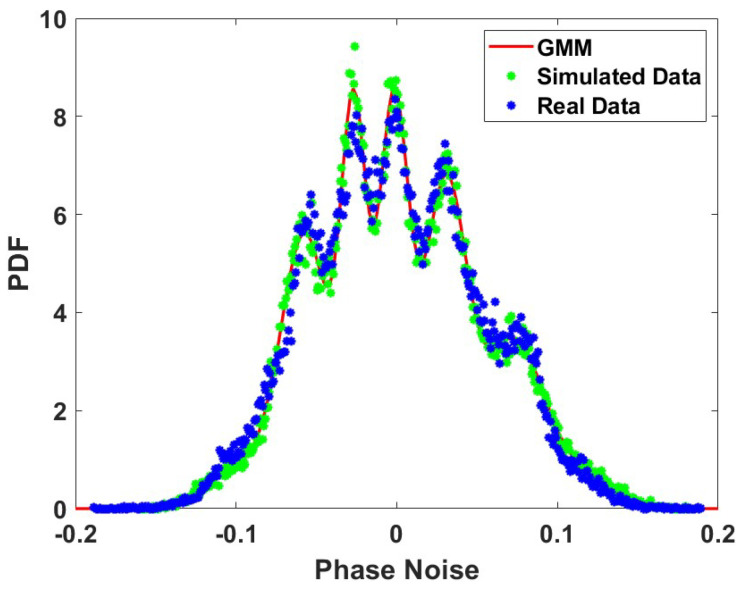
PDF of the simulated and experimental phase noise between WRLEN1 and WRLEN2 at T=1 s with 10 km of fibre.

This method of generating random variables requires an explicit expression for the CDF and its inverse [25]. However, in the case where no closed-form expression exists for the CDF, a numerical-based solution can be adopted instead. In the model adopted in this paper, the GMM model, no mathematical expressions exist for $F^{-1}$ and, therefore, we use the numerical method to computationally generate the random variables $\phi_{n}$ using the model parameters. The PDF of the simulated phase noise with the experimental phase noise is presented and compared in Figure 15; from the figure, we can observe that the model phase noise data closely match the experimental phase noise data.

### 6.2. Phase Noise Generation Using the Modelled ACF

The second and final step in generating the phase noise process, $\phi_{n}$, according to the developed models, is to manipulate the generated random process from the previous section to match the prescribed ACF. To create correlation in the IID random variables, we adopt the following process [26]:

**Step 2.1**: Generate a random variable matrix using a given PDF, following the outline in the previous section.

**Step 2.2**: Generate the covariance matrix with the desired correlation model.

**Step 2.3**: Decompose the covariance matrix in the triangular matrix by following the Cholesky decomposition.

**Step 2.4**: Multiply the random variable matrix with the Cholesky decomposition matrix to generate the correlated phase noise.

**Figure 16:**
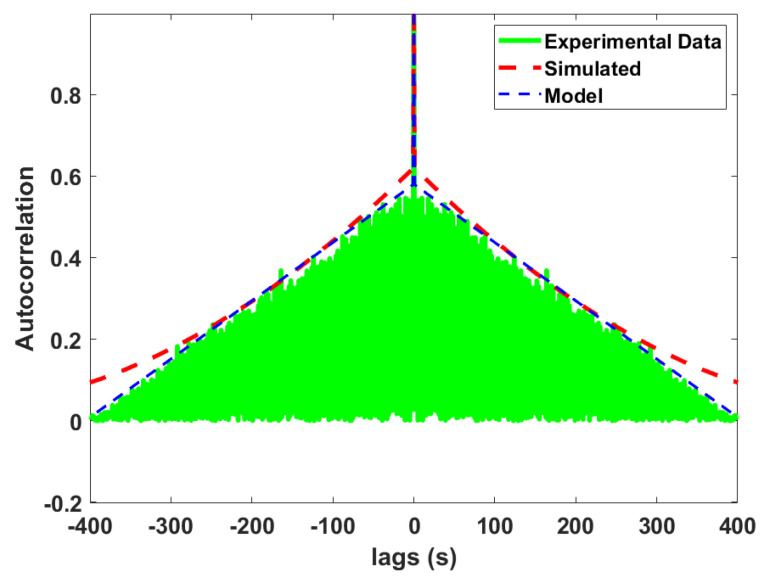
ACF of simulated and experimental phase noise between WRLEN1 and WRLEN2 at T=1 s with 10 km of fibre.

To verify the outcome, the model phase noise process is generated using the above-mentioned steps for data set 5 as an example, using the PDF and ACF model parameters for the corresponding data set. The ACF of the simulated and experimental phase noise is compared and shown in Figure 16; from the figure, we observe a good match for the ACF between the model phase noise data and the experimental phase noise data.

## 7. Conclusions

In this paper, we investigated the presence of phase noise processes in the time transfer over WR network-based optical fibre links. The interconnected nodes and devices contribute to the phase noise, and an accumulated effect is observed at different probing points in the WR network. We studied the statistical properties of the phase noise process present in time delay measurements over WRN-based optical fibre links and found that the probability density function of the phase noise process is not purely Gaussian in nature. It follows a Gaussian mixture model (GMM) with varying distribution parameters. Over a short period, at T=1 ms, peak-to-peak phase variations lie within 0.3 degrees for both test setups, i.e., test setup A with HROG-10 and test setup B with the testbed, and the distribution fits with a GMM model. The temperature sensitivity of the WR node configured in Grandmaster mode contributes to the increase in the number of mixing components in the GMM PDF if phase variations are recorded for a long period of time. The number of significant mixing components in the accumulated phase noise GMM PDF for the interconnected WRLEN1 and WRLEN3 through WRLEN2 are greater than the mixing components in the phase noise process recorded for individual links, i.e., between WRLEN1 and WRLEN2 and between WRLEN2 and WRLEN3. Furthermore, our phase noise process correlation study shows a significant correlation in phase noise at shorter lags, which slowly decreases at larger lags. The observed correlation trend is the same for all of the studied cases. However, the magnitude of the correlation coefficient at shorter lags differs for different cases.

After analysing and modelling the phase noise process, we verified the developed phase noise models by generating simulated phase noise processes using the developed models and comparing the simulated phase noise with the experimentally recorded phase noise process. The close match of simulated phase noise data with the experimental phase noise data confirms that the developed models are useful and effective for simulating the phase noise process. Therefore, these models would be quite helpful in developing phase noise reduction techniques and algorithms to compensate for phase offsets with low noise.

## Figures and Tables

**Table 1:** Gaussian mixture model parameters of different data sets for test setup A with HROG-10.

Data Sets	Optical Fibre Links	Mixing Component (K)	GMM Mean ($\mu_{i}$)	GMM Sigma ($\sigma_{i}$)	Component Proportion ($W_{i}$)
Data set 1: Phase offset between WRLEN1 and WRLEN2 at **T = 1 ms**	between WRLEN1 and WRLEN2 with a 10 km fibre	5	−0.0422385; −0.0366300; 0.0026105; 0.0396109; 0.0878236	0.0030000; 0.0008299; 0.0001102; 0.0001412; 0.0003211	0.010; 0.430; 0.280; 0.220; 0.060
Data set 2: Phase offset between WRLEN2 and WRLEN3 at **T = 1 ms**	between WRLEN2 and WRLEN3 with a 10 km fibre	5	−0.0906074; −0.0398586; 0.0076509; 0.0454425; 0.0929520	0.0001955; 0.0001685; 0.0001506; 0.0000892; 0.0004190	0.030; 0.320; 0.480; 0.130; 0.040
Data set 3: Phase offset between WRLEN1 and WRLEN3 at **T = 1 ms**	between WRLEN1 and WRLEN3 through WRLEN2 with 20 km fibres	8	−0.1293010; −0.0835311; −0.0421908; −0.0008504; 0.0493486; 0.0921653; 0.1305530; 0.1293010	0.0003055; 0.0002317; 0.0001715; 0.0002135; 0.0002798; 0.0003369; 0.0003994; 0.0004055	0.020; 0.170; 0.180; 0.220; 0.260; 0.110; 0.030; 0.010
Data set 4: Phase offset between WRLEN2 and WRLEN3 at **T = 1 s**	between WRLEN2 and WRLEN3 with a 10 km fibre	6	−0.0669749; −0.0739749; −0.0415965; −0.0092181; 0.0315547; 0.0711283	0.0018900; 0.0009905; 0.0008999; 0.0001819; 0.0001835; 0.0002911	0.010; 0.020; 0.340; 0.320; 0.240; 0.070
Data set 5: Phase offset between WRLEN1 and WRLEN2 at **T = 1 s**	between WRLEN1 and WRLEN2 with a 10 km fibre	7	−0.0986648; −0.0569917; −0.0266839; −0.0020589; 0.0301430; 0.0727634; 0.1172780	0.0003855; 0.0002292; 0.0000625; 0.0000870; 0.0001800; 0.0003519; 0.0003794	0.040; 0.210; 0.150; 0.190; 0.220; 0.160; 0.03

**Table 2:** Gaussian mixture model parameters of different data sets for test setup B with the testbed.

Data Sets	Optical Fibre Links	Mixing Component (K)	GMM Mean ($\mu_{i}$)	GMM Sigma ($\sigma_{i}$)	Component Proportion ($W_{i}$)
Data set 6: Phase offset between WRLEN1 and WRLEN2 at **T = 1 ms**	between WRLEN1 and WRLEN2 with 50 km fibre	8	−0.0933650; −0.0588505; −0.0358408; −0.0128311; 0.0170825; 0.0343380; 0.0596495; 0.0941641	0.0003211; 0.0002950; 0.0001987; 0.0001260; 0.0001282; 0.0001312; 0.0001982; 0.0003940;	0.048; 0.125; 0.115; 0.235; 0.197; 0.105; 0.120; 0.055;
Data set 7: Phase offset between WRLEN2 and WRLEN3 at **T = 1 ms**	between WRLEN2 and WRLEN3 with a 50 km fibre	8	−0.0981730; −0.0614800; −0.0362600; −0.0133300; 0.0118910; 0.0382600; 0.0588980; 0.0955860;	0.0003900; 0.0002650; 0.0001327; 0.0000897; 0.0000999; 0.0003319; 0.0003680; 0.0003940;	0.060; 0.120; 0.140; 0.170; 0.200; 0.220; 0.050; 0.040;
Data set 8: Phase offset between WRLEN1 and WRLEN3 at **T = 1 ms**	between WRLEN1 and WRLEN3 through WRLEN2 with a 100 km fibre	6	−0.1126410, −0.0764358; −0.0323600; 0.0101400; 0.0542160; 0.0951431;	0.0030000, 0.0004750; 0.0001616; 0.0001895; 0.0002499; 0.0001500;	0.040, 0.160; 0.240; 0.300; 0.200; 0.060;

**Table 3:** ACF model parameters for the phase noise process.

Data Set	Test Setup	Optical Fibre Links	a	b
Data set 1: Phase offset between WRLEN1 and WRLEN2 at **T = 1 ms**	A with HROG-10	between WRLEN1 and WRLEN2 with 10 km fibre	1200	0.36
Data set 2: Phase offset between WRLEN2 and WRLEN3 at **T = 1 ms**	A with HROG-10	between WRLEN2 and WRLEN3 with 10 km fibre	2300	0.20
Data set 3: Phase offset between WRLEN1 and WRLEN2 at **T = 1 ms**	A with HROG-10	between WRLEN1 and WRLEN3 with 20 km fibre	580	0.70
Data set 4: Phase offset between WRLEN2 and WRLEN3 at **T = 1 s**	A with HROG-10	between WRLEN2 and WRLEN3 with 10 km fibre	2200	0.20
Data set 5: Phase offset between WRLEN1 and WRLEN2 at **T = 1 s**	A with HROG-10	between WRLEN1 and WRLEN2 with 10 km fibre	700	0.58
Data set 6: Phase offset between WRLEN1 and WRLEN2 at **T = 1 ms**	B with the testbed	between WRLEN1 and WRLEN2 with 50 km fibre	930	0.46
Data set 7: Phase offset between WRLEN2 and WRLEN3 at **T = 1 ms**	B with the testbed	between WRLEN2 and WRLEN3 with 50 km fibre	930	0.46
Data set 8: Phase offset between WRLEN1 and WRLEN3 at **T = 1 ms**	B with the testbed	between WRLEN1 and WRLEN3 through WRLEN2 with 100 km fibre	930	0.46

## Data Availability

The data presented in this study are openly available in https://data.mendeley.com/datasets/9bssd95tn2/1.
